# ASO Author Reflections: Future of Reporting Complications After Gastric Cancer Surgery

**DOI:** 10.1245/s10434-025-17756-1

**Published:** 2025-07-01

**Authors:** Emilia Putila, Olli Helminen, Joonas H. Kauppila, on behalf of the FINEGO Group, Mika Helmiö, Heikki Huhta, Aapo Jalkanen, Anna Junttila, Raija Kallio, Vesa Koivukangas, Arto Kokkola, Elina Lietzen, Johanna Louhimo, Sanna Meriläinen, Vesa-Matti Pohjanen, Tuomo Rantanen, Ari Ristimäki, Jari Räsänen, Eero Sihvo, Vesa Toikkanen, Tuula Tyrväinen, Antti Valtola

**Affiliations:** 1 Surgery Research Unit, Medical Research Center Oulu, Oulu University Hospital and University of Oulu, Oulu, Finland; 2 Upper Gastrointestinal Surgery, Department of Molecular Medicine and Surgery, Karolinska Institutet and Karolinska University Hospital, Stockholm, Sweden

## PAST

Although the association of gastric cancer surgery with frequent occurrence of postoperative complications and high mortality is known, no current consensus exists on reporting postoperative complications after gastrectomy for gastric cancer.^1^ The lack of international consensus and standardization makes comparison between studies difficult. In 2015, the Esophagectomy Complications Consensus Group (ECCG) published a standardized list of complications after esophagectomy for esophageal cancer.^2^ The same list was later applied also for gastric cancer surgery^3^ because there are many surgical similarities between esophageal and gastric cancer. In 2019, the Gastrectomy Complications Consensus Group (GCCG) published a list of complications specific for gastrectomy.^4^ However, the applicability of these two classifications has not been compared in the context of gastric cancer surgery.

## PRESENT

This study aimed to compare the ECCG and GCCG classifications in reporting of postoperative complications after gastric cancer in a population-based setting. It is the first worldwide study comparing complication classifications in evaluating different types of complications in a gastric cancer context.^5^ For the 1115 patients in the current study, the occurrence of postoperative complications was 43% according to the ECCG classification versus 23% according to the GCCG classification. Most differences in reporting different types of postoperative complications were in relation to cardiac dysrhythmia (97% difference), infections (81% difference), and myocardial infarction (50% difference). For all three complications, the GCCG classification statistically reported a significantly lower incidence. Furthermore, 20 separate types (131 instances of occurrence) of postoperative complications after gastrectomy were detected using only the ECCG classification.

Regarding reoperations or reinterventions, the reported occurrence did not differ between the two classifications. The focus of the GCCG classification is on major complications, remarkably underestimating minor complications. However, because the GCCG classification often requires certain sequelae or treatments for complications, the occurrence of many major complications, such as strokes, also is remarkably underestimated compared with the ECCG classification.

## FUTURE

In light of the results from the current study, the ECCG classification appears to be the preferable classification system for reporting postoperative complications after gastric cancer surgery due to its comprehensiveness and simpler definitions of complications. The ECCG classification appears to be more sensitive than the GCCG classification for detecting the postoperative complications after gastrectomy for gastric cancer.

Establishing consensus and having one standardized list of complications after gastric cancer surgery would strongly benefit research by enabling not only comparison between studies, but also comparison of the complication profiles between other upper gastrointesinal tract surgeries. In the future, it would be beneficial to evaluate the applicability of these classifications in the context of benign upper gastrointestinal surgery. Furthermore, because some cancers in the esophagogastric junction are treated with either gastrectomy or esophagectomy, a harmonized classification for the two surgical approaches would benefit comparisons of surgical complications. An established consensus on reporting and identifying postoperative complications would improve the prevention and treatment of postoperative complications in the future.
